# Nanocrystalline SnO_2_ Functionalized with Ag(I) Organometallic Complexes as Materials for Low Temperature H_2_S Detection

**DOI:** 10.3390/ma14247778

**Published:** 2021-12-16

**Authors:** Timofei Goncharov, Abulkosim Nasriddinov, Anastasia Zubenko, Sergey Tokarev, Tatyana Shatalova, Nikolay Khmelevsky, Olga Fedorova, Marina Rumyantseva

**Affiliations:** 1 Faculty of Materials Science, Moscow State University, 119991 Moscow, Russia; goncharov.t.a@yandex.ru (T.G.); a.f.nasriddinov@gmail.com (A.N.); 2 Chemistry Department, Moscow State University, 119991 Moscow, Russia; shatalovatb@gmail.com; 3 A.N. Nesmeyanov Institute of Organoelement Compounds RAS, 119991 Moscow, Russia; nastya.mutasova@yandex.ru (A.Z.); pergeybokarev@gmail.com (S.T.); fedorova@ineos.ac.ru (O.F.); 4 LISM, Moscow State Technological University Stankin, 127055 Moscow, Russia; khmelevsky@mail.ru

**Keywords:** metal oxide gas sensor, nanocrystalline tin dioxide, Ag nanoparticles, Ag organometallic complexes, H_2_S sensor, low temperature detection

## Abstract

This paper presents a comparative analysis of H_2_S sensor properties of nanocrystalline SnO_2_ modified with Ag nanoparticles (AgNPs) as reference sample or Ag organic complexes (AgL1 and AgL2). New hybrid materials based on SnO_2_ and Ag(I) organometallic complexes were obtained. The microstructure, compositional characteristics and thermal stability of the composites were thoroughly studied by X-ray diffraction (XRD), X-ray fluorescent spectroscopy (XRF), Raman spectroscopy, Fourier transform infrared (FTIR) spectroscopy, X-ray photoelectron spectroscopy (XPS) and Thermogravimetric analysis (TGA). Gas sensor properties to 2 ppm H_2_S demonstrated high sensitivity, selectivity toward other reducing gases (H_2_ (20 ppm), NH_3_ (20 ppm) and CO (20 ppm)) and good reproducibility of the composites in H_2_S detection at low operating temperatures. The composite materials also showed a linear detection range in the concentration range of 0.12–2.00 ppm H_2_S even at room temperature. It was concluded that the predominant factors influencing the sensor properties and selectivity toward H_2_S in low temperature region are the structure of the modifier and the chemical state of silver. Thus, in the case of SnO_2_/AgNPs reference sample the chemical sensitization mechanism is more possible, while for SnO_2_/AgL1 and SnO_2_/AgL2 composites the electronic sensitization mechanism contributes more in gas sensor properties. The obtained results show that composites based on nanocrystalline SnO_2_ and Ag(I) organic complexes can enhance the selective detection of H_2_S.

## 1. Introduction

Hydrogen sulfide (H_2_S) is a colorless gas with an unpleasant smell of rotten eggs, causes severe corrosion of metals and is explosive in a mixture with air in the range of 4–45% vol. The main H_2_S sources are petroleum refining process, natural gas, geothermal sources, bacterial breakdown of organic matter and industrial activities [1]. Hydrogen sulfide is a toxic gas, affecting directly the human nervous and respiratory systems. People can sense H_2_S with concentrations of 0.005–0.3 ppm; however, at concentrations higher than 100 ppm the ability of smell might be lost, so H_2_S molecules dull the olfactory nerve and intoxication can occur unexpectedly [1,2]. According to the recommendation of National Institute for Occupational Safety and Health (NIOSH) of the USA the acceptable 10-min ceiling limit for H_2_S in workplace air must not exceed 10 ppm. However, an 8-h threshold limit value (TLV) of 1 ppm and a 15 min short-term exposure limit (STEL) of 5 ppm was recommended by the American Conference of Governmental Industrial Hygienists (ACGIH). World Health Organization recommends a guideline value of 0.15 mg/m^3^ (0.1 ppm) with an averaging time of 24 h [3].

The concentration of toxic gases, including hydrogen sulfide, can be monitored indoors and outdoors by different approaches [4]. Resistive type gas sensors based on wide-gap semiconductor oxides as a sensitive layer are promising devices for real-time monitoring of the environmental atmosphere and due to low cost, they can be commercially available to a wide variety of consumers [4,5,6]. However, one of the main disadvantages is their low selectivity. Different approaches were used in order to increase the selectivity and sensitivity to hydrogen sulfide by various scientific groups: composites based on n-type and p-type semiconductors [7,8,9,10,11,12], decoration and modification with catalytic phases and nanoparticles [13,14,15,16], the morphology change [17,18,19,20,21]. The use of organic-inorganic hybrid materials as gas sensors is a promisingly developing area and of particular interest [22,23,24]. Organometallic complexes being a receptor part of a gas sensor can reversibly react with the gas phase. Moreover, they may improve the adsorption of analyte molecules and form specific complexes [25]. The use of copper and silver as central cation can increase selective interaction with H_2_S, since these elements have a high affinity for sulfur, which can lead to significant decrease of the operating temperature [26,27].

This work is devoted to the study of the effect of Ag nanoparticles and silver organic complexes on the gas sensor properties of nanocrystalline tin dioxide in H_2_S detection. An enhanced sensitivity and selectivity to H_2_S was observed for composite materials in comparison to pristine SnO_2_ sensor. H_2_S sensing mechanism depending on the nature of modifier was discussed. The proposed composite materials are very promising for low temperature detection of H_2_S gas at low concentration level.

## 2. Materials and Methods

### 2.1. Material Synthesis

#### 2.1.1. Synthesis of Nanocrystalline SnO_2_

Nanocrystalline SnO_2_ was obtained by chemical precipitation method at the room temperature. The precursor SnCl_4_⋅5H_2_O (10.00 g, 98%, Sigma-Aldrich, St. Louis, MO, USA) was dissolved in distilled water (100 mL). Then aqueous ammonia (1 M) was added dropwise to the solution while stirring with a magnetic stirrer until pH ≈ 7. The obtained precipitate was separated from the solution by centrifugation (3600 rpm, 3 min.), then was thoroughly washed with 0.01 M solution of NH_4_NO_3_ (99%, Sigma-Aldrich) until complete purification from chloride anions (test by the reaction with AgNO_3_). Final washing was carried out with deionized water. The resulting pure precipitate of the α-stannic acid gel was dried in drying chamber at 80 °C for 24 h. Final product was ground in an agate mortar and then annealed at 300 °C for 24 h.

#### 2.1.2. Synthesis of Ag Organic Complexes

**Figure 1:**
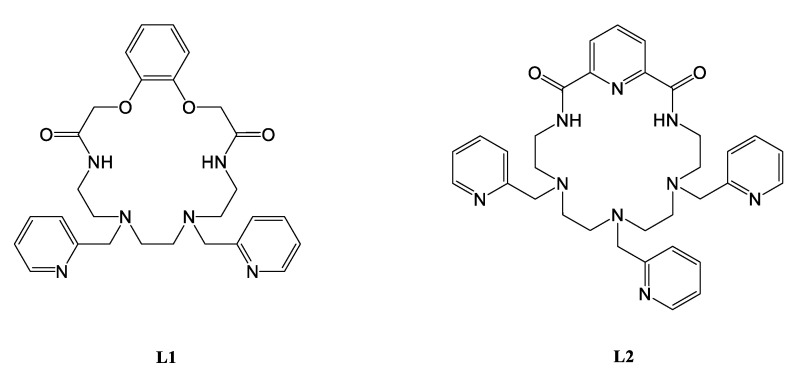
The structures of L1 and L2 ligands.

All commercially available reagents and solvents were used without further purification. Ligands L1 and L2 (Figure 1) were prepared as described earlier [28,29].

Complex [Ag_2_L1](NO_3_)_2_·2H_2_O: The solution of AgNO_3_ (14 mg, 0.081 mmol) in H_2_O (2 mL) was added to the solution of L1 (21 mg, 0.0405 mmol) in MeOH (1 mL), and the mixture was kept at room temperature without stirring for 5 h. After the formation of a brown precipitate, the solution was decanted and evaporated in vacuo. The residue was recrystallized from ethanol to obtain the complex. The complex yield was 60% and was shortly named as AgL1.

Complex [Ag_3_L2](NO_3_)_3_·4H_2_O: The solution of AgNO_3_ (18 mg, 0.1061 mmol) in H_2_O (2 mL) was added to the solution of L2 (21 mg, 0.03537 mmol) in MeOH (2.5 mL), and the mixture was kept at room temperature without stirring for 6 h. The residue was recrystallized from ethanol to obtain the complex. The complex yield was 50 % and was shortly named as AgL2.

#### 2.1.3. Synthesis of Composite Materials

Composites based on nanocrystalline SnO_2_ and Ag nanoparticles (AgNPs) or Ag organic complexes were obtained by impregnation method. Ag complexes and AgNO_3_ were dissolved in distilled water and added to the powder of SnO_2_ in portions of 10 μL. After adding each portion, the mixture was thoroughly mixed and completely dried until the solvent evaporated in a drying chamber at a temperature of 60 °C. This procedure was repeated until the composite was completely prepared. SnO_2_ impregnated with AgNO_3_ solution, was additionally annealed at 300 °C for 24 h. The amount of the introduced modifier was calculated so that the Ag content in the composites was 1 at.%. The obtained composite materials were named as SnO_2_/AgNPs, SnO_2_/AgL1 and SnO_2_/AgL2, respectively.

### 2.2. Materials Characterization

The phase composition of the nanocrystalline SnO_2_ was characterized by X-ray powder diffraction (XRD) using DRON-4 diffractometer (Burevestnik, Moscow, Russia) with CuK_α1_ radiation (λ = 1.54059 Å) and Raman spectroscopy method. Raman spectra were recorded on i-Raman Plus (BW Tek, Newark, DE, USA) spectrometer equipped with a BAC 151C microscope in the range of 90–3500 cm^−1^ with a resolution of 4 cm^−1^. A green laser with λ = 532 nm was used as a radiation source. The measurement of the specific surface area of SnO_2_ was carried out by low-temperature nitrogen adsorption on Chemisorb 2750 (Micromeritics) equipment using the BET model (Brunauer, Emmett, Teller).

Elemental analysis of the Ag complexes was carried out on a Carlo Erba 1108 elemental analyzer. Electrospray ionization mass spectrometry (ESI-MS) analysis was performed using a Finnigan LCQ Advantage mass spectrometer equipped with an octopole ion-trap mass-analyzer, an MS Surveyor pump, a Surveyor auto sampler, and a Schmidlin-Lab nitrogen generator. ^1^H NMR spectra were recorded at 25 °C on Varian Inova 400 MHz spectrometer. Chemical shifts were reported in parts per million (*δ)* relative to deuterated solvent as an internal reference.

The elemental composition of composite materials was investigated on M1 Mistral X-ray fluorescent spectrometer (Bruker Nano GmbH, Berlin, Germany) with the beam energy of 50 keV. X-ray photoelectron spectra (XPS) were performed on the XPS system (Thermo Fisher Scientific, Waltham, MA, USA) equipped with a hemispherical analyzer and using monochromatic Al $K_{\alpha }$ radiation as X-ray source (1486.7 eV). Fourier transform infrared (FTIR) spectroscopy was conducted to investigate the structural fragments of Ag complexes on the surface of semiconductor tin dioxide. FTIR spectra were recorded from KBr pellets (50.0 mg) mixed with the test sample (0.3–0.5 mg) on Perkin Elmer Spectrum One spectrometer in the transmission mode in the range of 4000–400 cm^−1^ with a resolution of 1 cm^−1^. Thermogravimetric analysis was carried out on a NETZSCH STA 449 device (Netzsch-Gerätebau GmbH, Selb, Germany ) combined with a QMS-409 mass spectrometer, which was used to determine the range of thermal stability of the obtained composites. The samples were heated in air current (30 mL/min) up to 500 °C at a heating rate of 10 °C/min.

Micro-hotplates with Pt-electrodes were used for gas sensing tests. The powders of composites were mixed with ethanol to form a paste and then deposited in a form of thick film on the surface of hotplates. The films were dried at 60 °C for 24 h and were annealed at 150 °C for 3 h with heating rate of 1 °C/min.

The gas sensing experiments were performed in laboratory-equipped electronic module at the temperature range of 150–25 °C. The sensors were exposed to various concentrations of H_2_S gas in the range of 0.12–2 ppm, the concentration was controlled by mass flow controllers. The flow of the analyzed gas was alternated with purified air at an interval of 30 min. The ratio of the resistance in pure air (R_air_) and in detecting gas (R_gas_) was defined as the gas sensor signal S = R_air_/R_gas_.

## 3. Results and Discussion

### 3.1. Characteristics of Nanocrystalline SnO_2_

**Figure 2:**
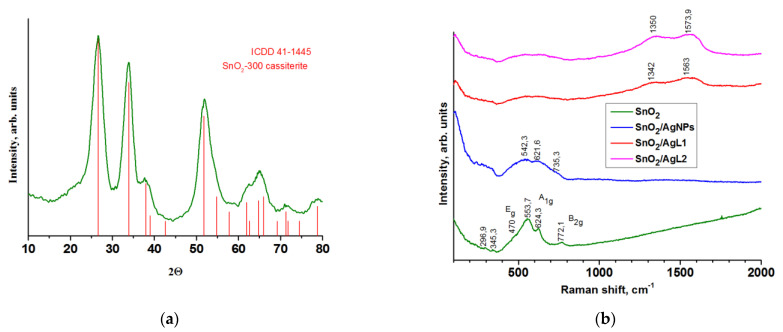
XRD pattern of SnO_2_ (**a**) and Raman spectra of composites and pure SnO_2_ (**b**).

XRD analysis established that obtained nanocrystalline SnO_2_ is single phase. The XRD pattern corresponds to the tetragonal cassiterite structure (ICDD 41–1445) (Figure 2a). The average crystallites size calculated from the broadening of the most intense peaks using Scherer equation was equal to 3–4 nm. The specific surface area of nanocrystalline tin dioxide is 138 ± 5 m^2^/g.

The Raman spectrum of SnO_2_ is shown at Figure 2b. Tetragonal rutile structure of SnO_2_ is described by three characteristic vibrational modes $E_{g}$ (470.0 cm^−1^), $A_{1g}$ (624.3 cm^−1^) and $B_{2g}$ (772.1 cm^−1^). The A_1g_ and B_2g_ modes are related to symmetric and asymmetric Sn–O stretching orthogonally to the *c*-axis, respectively. The $E_{g}$ mode is attributed to the motion of O anions along the *c*-axis [30,31]. The widest band at 553.7 cm^−1^ is associated with in-plane oxygen vacancies of the nanocrystalline SnO_2_ [32,33]. The bands at 296.9 and 345.3 cm^−1^ are related to the $E_{u}$ mode and associated with transformation of an IR to Raman active modes [34].

### 3.2. Characteristics of Composite Materials

The ^1^H NMR and ESI-MS spectra for AgL1 and AgL2 complexes are shown in Figures S1–S4, respectively. The results of elemental analysis showed that the composition of the ligand to metal is (1:2) for AgL1 and (1:3) for AgL2 complexes, respectively. At the same time, there is only one (1:1) complex for AgL1 and there are two complexes (1:1) and (1:2) (ligand: metal) for AgL2 according to ESI-MS analysis. During recording procedure of the ESI-MS spectra, the test sample heats up to about 120–150 °C. It can be assumed, that at this temperature, the complex with the composition of the ligand to metal (1:2) still exists in the AgL2 sample, but in the AgL1 it seems to have fallen apart. Therefore, up to 100 °C AgL1 is present on the surface in the form of a (1:2) complex (maybe partially (1:1)), and after 100 °C, only (1:1) remains. AgL2 is present up to 100 °C in the form of (1:3) (with possible impurities of (1:2) and (1:1)), which decomposes to (1:2) with increasing temperature. That is why, the (1:2) complex of the AgL2 is more stable than that of the AgL1.

The elemental composition of the surface of composite materials based on nanocrystalline SnO_2_ is shown in Table 1. It can be seen that the silver content in the composites is close to the set value of [Ag]/([Ag] + [Sn]) = 1 at.%. These values were averaged, taken from three different areas of the sample. At the same time, the primary values were also close to each other, which indicates a uniform distribution of the Ag complex molecules and AgNPs on the surface and effective sensitization.

Composite materials SnO_2_/AgNPs, SnO_2_/AgL1 and SnO_2_/AgL2 were studied using Raman spectroscopy (Figure 2b). The low-frequency region in composites arises from vibration modes of SnO_2_ nanoparticles. The Raman spectra of SnO_2_/AgL1 and SnO_2_/AgL2 contain wide bands, at frequencies of 1342 cm^−1^, 1563 cm^−1^ and 1350 cm^−1^, 1574 cm^−1^, respectively. The bands at 1563 cm^−1^ and 1573.9 cm^−1^ are assigned to the symmetric stretching vibration of the C=C bonds in Py and phenyl (in case of L1) rings [35]. The bands near 1350 cm^−1^ are associated with the vibration of the C=C and C=N bonds of the Py rings [36,37]. Raman bands observed near 1300–1180 cm^−1^ are due to in-plane deformations vibrations of ring C–H bond [38,39]. A decrease in the intensity of the bands related to tin dioxide indicates its predominant surface coating with Ag organic complexes, while for AgNPs these peaks are much more intense.

**Figure 3:**
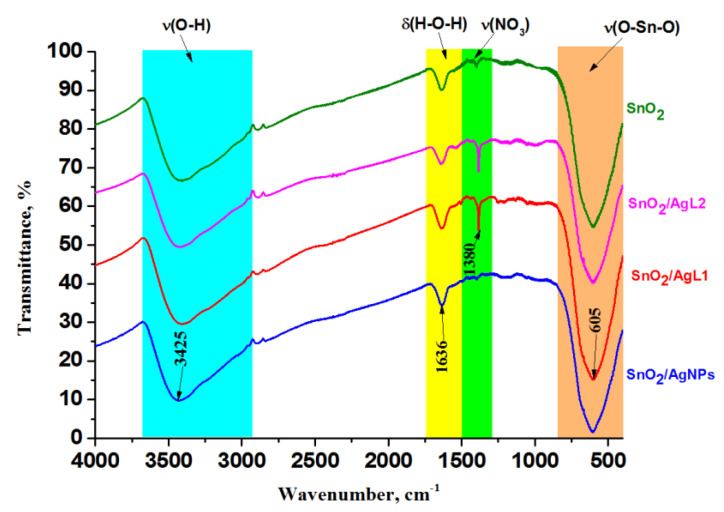
FTIR spectra of pure SnO_2_ and composites.

Figure 3 represents the FTIR spectra of composite materials. The wide band at 605 cm^−1^ corresponding to stretching vibrations of Sn–O bonds is present on all spectra [40,41]. The wide band with a peak at 3425 cm^−1^ at the region of 3600–3000 cm^−1^ and sharp band at 1636 cm^−1^ indicate the presence of adsorbed water, which refers to the OH valence and stretching vibrations, respectively [41]. A vibrational mode corresponding to the nitrate groups is observed at the frequency of 1380 cm^−1^ [41]. This is due to the fact that during synthesis, tin dioxide was washed with ammonium nitrate to remove chloride ions and subsequently nitrate ions remain on the sample in small quantities. However, it can be observed that the intensity of the NO_3_^−^ ion vibrational mode is quite high in complexes with aza-crown compounds, and this is directly related to the presence of a nitrate counter ion in the composition of the complexes.

**Figure 4:**
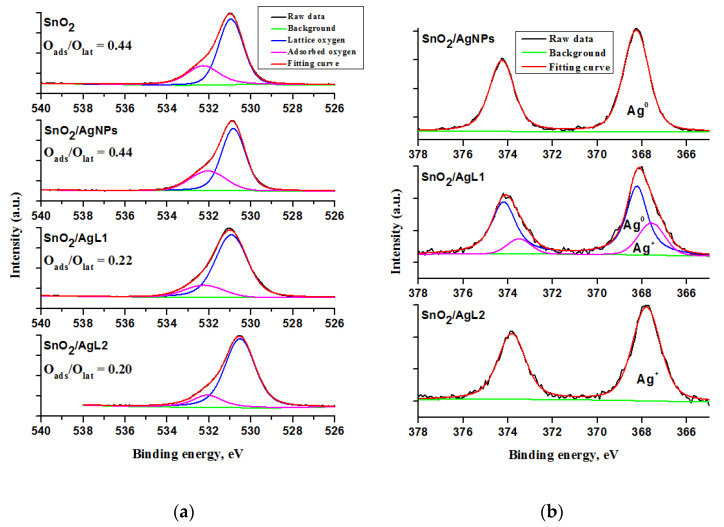
X-ray photoelectron spectra of the pure SnO_2_ and composite materials for O1s (**a**) and Ag3d (**b**) core level binding energies.

The XP spectra of the pure SnO_2_ and composite materials are shown in Figure 4. Two components of the O1s spectra locating at 530.9–530.5 eV and 532.0–532.2 eV are attributed to lattice oxygen (O_lat_) and adsorbed (O_ads_) oxygen species/hydroxyl ions (O^−^, O^2−^ and OH^−^), respectively [42].

The deconvolution of the Ag3d spectrum of the SnO_2_/AgL1 composite showed that silver is present in both metallic state Ag^0^ (368.3 eV) and oxidized state Ag^+^ (367.6 eV) that indicates a partial reduction of silver in sensitization process. However, the binding energy of 367.7 eV of the Ag3d spectrum of the SnO_2_/AgL2 composite is assigned only to Ag^+^, while AgNPs are attributed to metallic state (368.2 eV) [42,43,44].

Silver in organic complexes should be in the chemical state of +1. According to the ^1^H NMR and ESI-MS analysis for AgL1 and AgL2 complexes and its discussion above, it was concluded, that the (1:2) complex of the AgL2 is more stable than that of the AgL1. Moreover, cyclic voltammogram analysis showed that there are a lot of free or weakly bounded silver cations in AgL1 and AgL2 complexes (Figures S5–S7). Therefore, it can be assumed that the partial reduction of silver in the AgL1 complex may be a consequence of the interaction with the metal oxide matrix.

**Figure 5:**
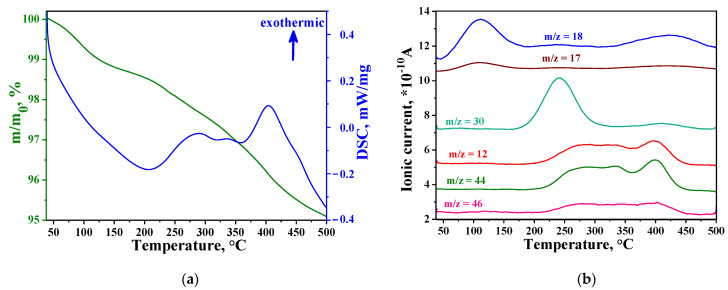
Thermal analysis (**a**) and mass spectrometry curves (**b**) of SnO_2_/AgL1 composite.

The results of thermogravimetric analysis (TGA) for SnO_2_/AgL1 composite are shown in Figure 5. TGA and MS data for the other composite have similar character. The analysis showed that in the temperature range of 50–175°C, evaporation of adsorbed water occurs, which is proven by an increase in the ionic current associated with H_2_O (m/z = 17, m/z = 18). Aza-crown compounds begin to decompose at a temperature of 180–200 °C, as evidenced by the release of CO_2_ (m/z = 12, m/z = 44) and NO (m/z = 30), accompanied by an exothermic effect.

### 3.3. Gas Sensor Properties

**Figure 6:**
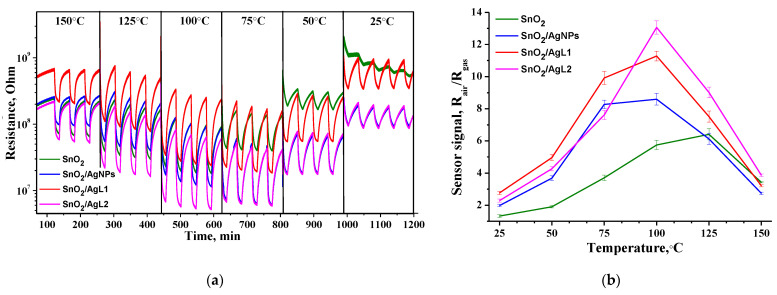
(**a**) The temperature dependence of the sensors’ resistance of composite materials on the periodical change of the gas phase composition from pure air to 2 ppm H_2_S in air at different temperatures; (**b**) Dependence of the sensor signal on temperature at a constant concentration of 2 ppm H_2_S.

Figure 6a represents the temperature dependence of the sensors’ resistance on a dynamic change of the gas phase composition (30 min–pure air, 15 min–2 ppm H_2_S in air). The study of sensor properties at the constant concentration of 2 ppm H_2_S was performed from 150 °C to 25 °C, since heating above 150 °C will lead to thermal destruction of organic compounds according to TGA results. All sensors exhibit n-type behavior: resistance decreases during exposure to the reducing gas (H_2_S) and returns to initial value during pure air pulse. A decrease in the operating temperature leads to the increase in the baseline resistance. Moreover, in contrast to pristine SnO_2_, composite materials exhibit a stable and well reversible sensor signal.

The analysis of the temperature dependences of sensor signal (Figure 6b) showed that sensitization of tin dioxide with Ag organic complexes leads to the appearance of a sensor signal at low temperatures. The temperature of the maximum sensor signal decreases down to 100 °C, and the sensor signal itself increases more than 2 times, in comparison to pristine SnO_2_, which exhibits a significant signal at a higher temperature.

The nature of the sensor signal is associated with interaction between chemisorbed oxygen species with reducing gas and is based on surface-depletion model [45]. In pure air atmosphere the oxygen molecules adsorb on and interact with the SnO_2_ surface by transferring electrons from the conduction band (reactions 1–2). The resulting depletion layer leads to an increase in the resistance of the material (Figure 6a). The predominant oxygen ionic species will be O_2_^-^, since the operating temperature is above 150 °C [45,46]. The resistance of the sensors decreases due to the reaction of H_2_S molecules with chemisorbed oxygen (reaction 3); electrons localized on chemisorbed oxygen are released, pass into the conduction band of the semiconductor, which leads to a decrease in the electrical resistance of the samples.

(1) $$O_{2\left(\mathrm{gas}\right)}\;\to {\;O}_{2\left(\mathrm{ads}\right)}$$

(2) $$O_{2\left(\mathrm{ads}\right)}+{\;e}^{-}\;\to {\;O}_{2\left(\mathrm{ads}\right)}^{-}$$

(3) $$\beta \cdot H_{2}S_{\left(\mathrm{gas}\right)}+3O_{\beta }^{-\alpha }\;\to \;\beta \cdot {\mathrm{SO}}_{2\left(\mathrm{gas}\right)}+\;\beta \cdot H_{2}O_{\left(\mathrm{gas}\right)}+3\alpha \cdot e^{-}$$

**Figure 7:**
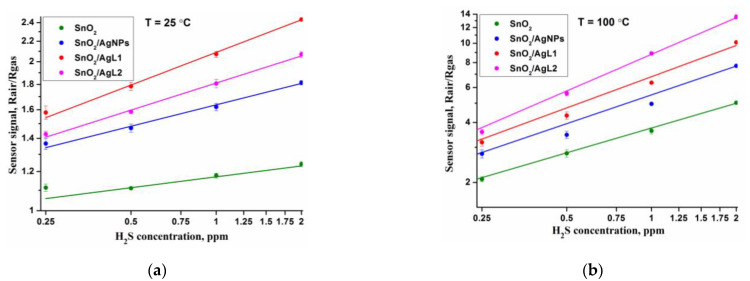
The dependence of the sensor signal on H_2_S concentration at constant temperatures of 25 °C (**a**) and 100 °C (**b**).

Sensor properties of the obtained nanocrystalline SnO_2_ and composite materials were also studied in the concentration range of 0.12–2.00 ppm H_2_S in dry air. The measurements were carried out with an increase of the concentration of the target gas. Figure 7 shows the dependence of the sensor signal on H_2_S concentration at constant temperatures of 25 °C and 100 °C, which is well linearized in double logarithmic coordinates. In both cases composite materials exhibit enhanced sensor response. The modified composites are able to detect H_2_S even at room temperature in sub-ppm concentration range.

**Figure 8:**
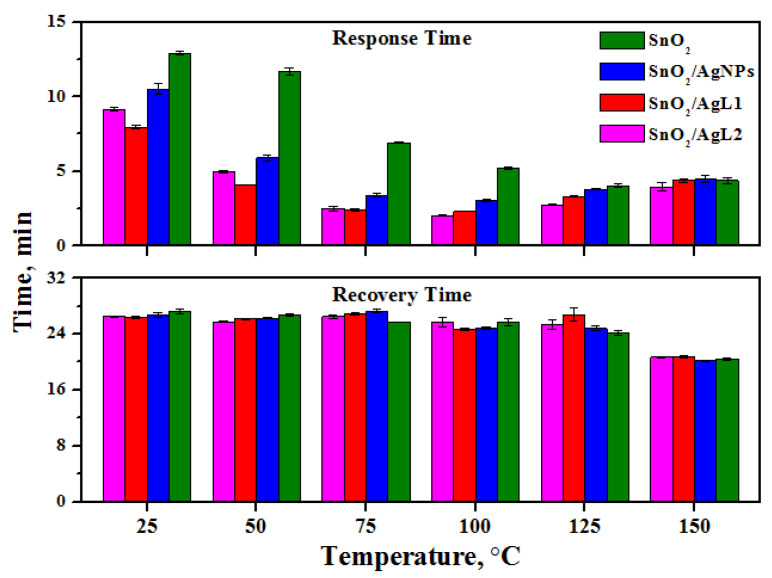
Response time and recovery time of nanocrystalline SnO_2_ and composite materials toward exposure to 2 ppm H_2_S gas at the temperature range of 25–150 °C.

The response time (t_res_) was determined as the time required to reach 90% of the maximum sensor signal during exposure to 2 ppm H_2_S in 15 min and the recovery time (t_rec_) was determined as the time required for 90% of the sensor response change after removal of the H_2_S from the gas phase and during exposure to pure air in 30 min (Figure 8). This method of measuring the t_res_ and t_rec_, which is slightly differ from the traditional one when saturation is fully reached, was used for a comparative analysis of the kinetics of the interaction of analyte-gas molecules with samples under specified conditions. It can be seen that the response time values correlate with the values of the sensor signal for all samples in the entire temperature range, which indicates the importance of the kinetic component of the interaction of the material with the gas phase. The shorter the response time, the faster the reaction proceeds and, accordingly, the higher the sensor signal. However, at each temperature, the recovery time is approximately the same for all samples.

**Figure 9:**
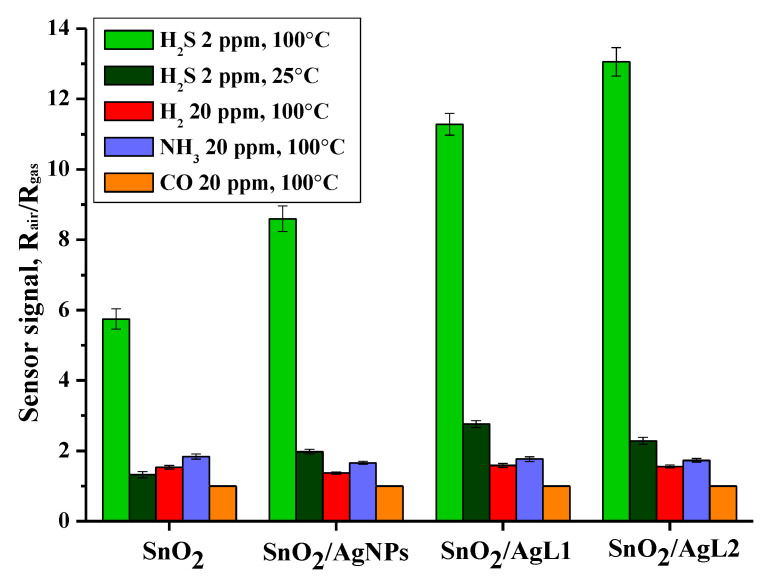
Sensor signal toward H_2_S (2 ppm), H_2_ (20 ppm), NH_3_ (20 ppm) and CO (20 ppm) for different samples.

Selectivity of the samples was investigated toward four different reducing gases–H_2_S (2 ppm), H_2_ (20 ppm), NH_3_ (20 ppm) and CO (20 ppm) (Figure 9). The temperature at which the maximum sensor signal to H_2_S was observed is 100 °C, therefore, the selectivity toward interfering gases was investigated also at 100 °C. Moreover, the composites showed a significant sensor signal at room temperature only for H_2_S. It can be seen that composite materials selectively detect H_2_S even at room temperature, while the temperature of the maximum sensor signal for other gases was much higher, and their concentration was 10 times higher.

The enhanced response of the composite materials to H_2_S gas in comparison to pristine SnO_2_ might be due to several factors. On the one hand, one could assume the above-mentioned mechanism based on H_2_S interaction with chemisorbed oxygen, but this mechanism is not able to explain the selectivity enhancement. On the other hand, the results obtained by the XPS method indicate that the concentration of chemisorbed oxygen is 2 times higher in pristine SnO_2_ and SnO_2_/AgNPs composite in comparison to SnO_2_/AgL1 and SnO_2_/AgL2 composites (Figure 4a). However, the sensor signal of the latter, on the contrary, is 2 times higher than that of the pristine SnO_2_ and SnO_2_/AgNPs composite. Comparing XPS results with sensor measurements, we can conclude that the predominant factors influencing the sensor properties and selectivity toward hydrogen sulfide in low temperature region are the structure of the modifier and the chemical state of silver in it.

SnO_2_/AgNPs composite contains metallic silver nanoparticles dispersed on SnO_2_ surface. In this case, Ag NPs act as catalytic additives; therefore, chemical sensitization is more likely. After dissociative adsorption of the oxygen molecules on the surface of AgNPs their spillover on the SnO_2_ surface is occurs. The produced oxygen species can actively oxidize H_2_S molecules, leading to the increase in sensor response [47].

The situation with SnO_2_/AgL1 and SnO_2_/AgL2 composites is slightly different, since there is also Ag^+^. Therefore, the electronic sensitization mechanism is typical for them, which involves the exchange of electrons between the modifier and the nanocrystalline semiconductor. The electronic potential of the redox couple Ag^+^/Ag^0^ is 5.3 eV below the vacuum level. The energy band of SnO_2_ will be bent until the Fermi energy of SnO_2_ (4.9 eV) is pinned at the energy level of this redox couple in order to achieve electronic equilibrium. As a result the electron-depleted region is emerged. In the atmosphere of H_2_S (reducing gas) the equilibrium of Ag^+^/Ag^0^ is more likely shifted to Ag^0^. Therefore, E_F_(SnO_2_) is pinned at the level of the Ag work function (4.5 eV), as a result the conductivity of SnO_2_ will be increased. Matsushima et al. have showed that the electronic sensitization mechanism contributes more in gas sensor properties of the Ag-SnO_2_ system than the chemical sensitization mechanism [48]. That’s why SnO_2_/AgL1 and SnO_2_/AgL2 composites show higher sensor response to H_2_S in comparison to SnO_2_/AgNPs composite.

On the other hand, SnO_2_/AgL1 and SnO_2_/AgL2 composites have different sensor responses toward H_2_S. It can be observed from the Figure 6b, that SnO_2_/AgL1 composite has higher response up to 100 °C; however, above 100 °C SnO_2_/AgL2 composite shows the best results among the samples. As it was mentioned above, up to 100 °C AgL1 is present in the form of a (1:2) complex (maybe partially (1:1)), and after 100 °C, only 1:1 remains. AgL2 is present up to 100 °C in the form of (1:3) (with possible impurities of (1:2) and (1:1)), which decomposes to (1:2) with increasing temperature. It means, that the complex with (1:2) composition is more preferable for H_2_S detection. This may be due to steric effects that allow silver in this complex to reversibly change its oxidation state depending on the environment. Ag has a high affinity for sulfur. When H_2_S molecules interact with silver, a bridging bond can be formed in which one sulfur atom is attached to two silver atoms. Therefore, in this case, the close location of two silver atoms in the complex (1:2) will be more advantageous for increasing the adsorption energy during the formation of a bond with sulfur (reaction 4) [49].

(4) $$H_{2}S_{\left(\mathrm{ads}\right)}+2{\mathrm{Ag}}_{\left(s\right)}+\frac{1}{2}\;O_{2\left(\mathrm{ads}\right)}^{-}\;\to {\mathrm{Ag}}_{2}S_{\left(s\right)}+{\;H}_{2}O_{\left(\mathrm{gas}\right)}+{\;e}^{-}$$

## 4. Conclusions

In summary, highly selective and sensitive materials were obtained for H_2_S detection at low operating temperatures. SnO_2_ was used as a matrix of the sensitive layer and was prepared by chemical precipitation method. Ag nanoparticles (AgNPs) or Ag organic complexes (AgL1 and AgL2) were used as surface modifiers. Modified composites were obtained by impregnation method.

Sensing performance was studied in the temperature range of 150–25 °C. It was found that modification with Ag organic complexes leads to shift of the operating temperature vs. lower temperature region and sensor signal to H_2_S was increased more than two times. It was observed that the response times exactly coincide with the values of the sensor signal for all samples in the entire temperature range that indicates the importance of the kinetic component of interaction of the material with the gas phase. The modified composites are able to detect H_2_S at sub-ppm concentration level even at room temperature. Selectivity of the sensors was tested towards four types of reducing gases. The results showed a highly selective behavior of the composites to H_2_S.

It was concluded that the predominant factors influencing the sensor properties and selectivity toward H_2_S in low temperature region are the structure of the modifier and the chemical state of silver. Thus, in the case of SnO_2_/AgNPs reference sample, the chemical sensitization mechanism is more possible, while for SnO_2_/AgL1 and SnO_2_/AgL2 composites the electronic sensitization mechanism contributes more in gas sensor properties.

## Figures and Tables

**Table 1:** The elemental composition of materials.

Sample	SnO_2_/AgNPs	SnO_2_/AgL1	SnO_2_/AgL2
[Ag]/([Ag] + [Sn]), at.%	1.06 ± 0.01	1.01 ± 0.01	0.98 ± 0.01
[Sn]/([Ag] + [Sn]), at.%	98.94 ± 0.10	98.99 ± 0.10	99.02 ± 0.10

## Data Availability

The data presented in this study are available upon request from the corresponding author. The data are not publicly available due to privacy reasons.

## Supplementary Materials

The following are available online at https://www.mdpi.com/article/10.3390/ma14247778/s1, Figure S1: ESI-MS spectrum of the complex of the ligand L1 with Ag^+^ in water, Figure S2: ^1^H spectrum of the complex of the ligand L1 with Ag^+^ in D_2_O, Figure S3: ESI-MS spectrum of the complex of the ligand L2 with Ag^+^ in water, Figure S4: ^1^H spectrum of the complex of the ligand L2 with Ag^+^ in D_2_O, Figure S5: Cyclic voltammogram of AgL1 complex, Figure S6: Cyclic voltammogram of AgL2 complex, Figure S7: Cyclic voltammogram of AgClO_4_.
