# Supplementary material for: Nanocrystalline SnO2 Functionalized with Ag(I) Organometallic Complexes as Materials for Low Temperature H2S Detection
Source: Materials (Basel). 2021 Dec 16;14(24):7778. doi: 10.3390/ma14247778 (PMC8707773; doi:10.3390/ma14247778)
Supplement: Supplementary file 1 [file materials-14-07778-s001.zip › materials-1506131-supplementary.pdf]

materials

Supplementary Materials

Nanocrystalline SnO_2 Functionalized with Ag(I) Organometallic Complexes as Materials for Low Temperature H_2S Detection

Timofei Goncharov [1], Abulkosim Nasriddinov [1,2], Anastasia Zubenko [3], Sergey Tokarev [3], Tatyana Shatalova [2], Nikolay Khmelevsky [4], Olga Fedorova [3] and Marina Rumyantseva [2,*]

[1] Faculty of Materials Science, Moscow State University, 119991 Moscow, Russia; goncharov.t.a@yandex.ru (T.G.); a.f.nasriddinov@gmail.com (A.N.)
[2] Chemistry Department, Moscow State University, 119991 Moscow, Russia; shatalovatb@gmail.com
[3] A.N. Nesmeyanov Institute of Organoelement Compounds RAS, 119991 Moscow, Russia; nastya.mutasova@yandex.ru (A.Z.); pergeybokarev@gmail.com (S.T.); fedorova@ineos.ac.ru (O.F.)
[4] LISM, Moscow State Technological University Stankin, 127055 Moscow, Russia; khmelevsky@mail.ru
* Correspondence: roum@inorg.chem.msu.ru; Tel.: +7-495-939-5471

Elemental analysis, calculated for $C_{28}H_{38}Ag_2N_8O_{12}$ ($[Ag_2C_{28}H_{34}N_6O_2](NO_3)_2 \cdot 2H_2O$): C, 37.60; H, 4.28; N, 12.53, found: C, 37.49; H, 4.19; N, 12.44 (Figure S1). ESI-MS, *m*/*z*: calculated for $C_{28}H_{34}N_6O_4$+Ag^+: 625.2 $[L1+Ag]^+$; found: 625.9 (Figure S2).

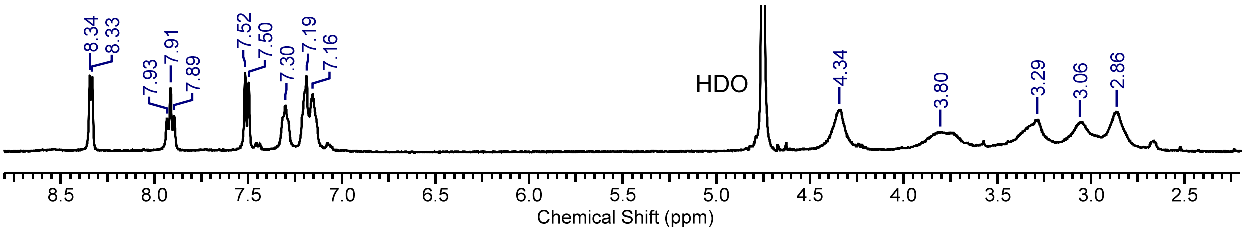

Figure S1. ^{1}H spectrum of the complex of the ligand **L1** with Ag^+ in D_2O.

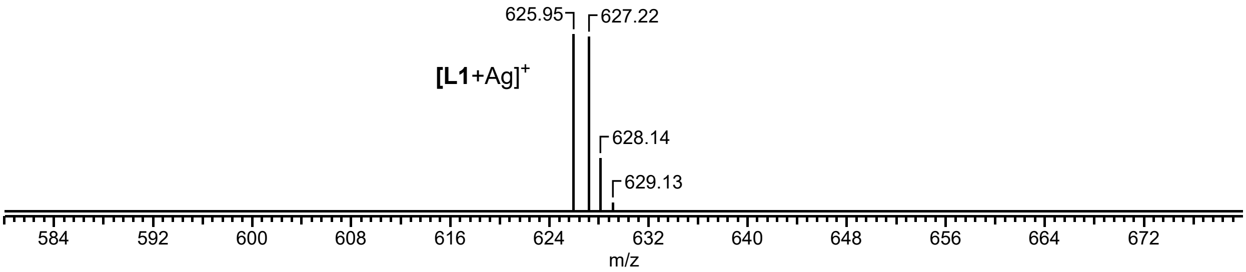

Figure S2. ESI-MS spectrum of the complex of the ligand **L1** with Ag^+ in water.

Elemental analysis, calculated for $C_{33}H_{47}Ag_3N_{12}O_{15}$ ($[Ag_3C_{33}H_{39}N_9O_2](NO_3)_3 \cdot 4H_2O$): C, 33.72; H, 4.03; N, 14.30, found: C, 33.54; H, 3.91; N, 14.21 (Figure S3). ESI-MS, *m*/*z*: calculated for $C_{33}H_{39}N_9O_2$+Ag $^+$: 700.2 $[L2+Ag]^+$; found: 700.6; calculated for $C_{33}H_{38}N_9O_2$+2Ag^+: 806.1 $[L2-H+2Ag]^+$; found: 806.1 (Figure S4).

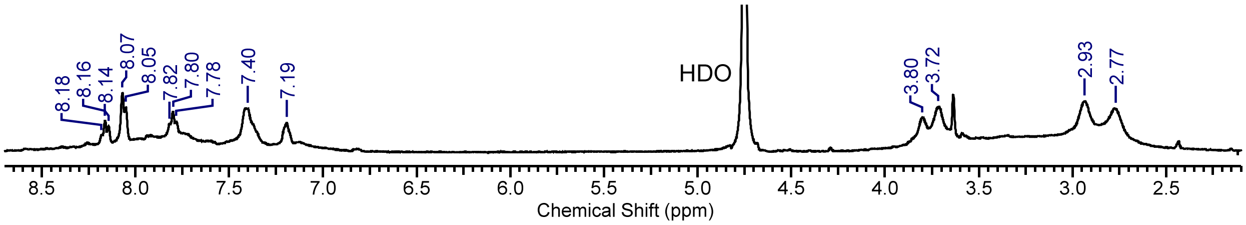

Figure S3. ^{1}H spectrum of the complex of the ligand **L2** with Ag^{+} in D_2O.

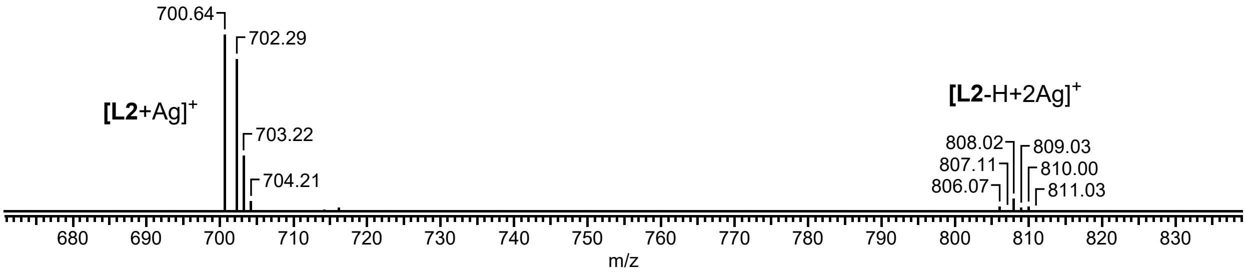

Figure S4. ESI-MS spectrum of the complex of the ligand **L2** with Ag^{+} in water.

Electrochemical measurements were carried out at 22°C with a Metrohm Autolab B.V. potentiostat type: PGSTAT128N. Cyclic voltammetry experiments were performed in three-electrode cell equipped with a glassy carbon (GC) working electrode (disk, *d*=2 mm), Ag/AgCl/KCl (aq saturated) reference electrode, and platinum counter electrode. Compounds were dissolved (10^{-3} M) in degassed dry acetonitrile containing TBAHFP as the supporting electrolyte (0.1 M). Dry argon gas was bubbled through the solutions for 10 min before cyclic voltammetry experiments. The applied scan rate was 100 mV s^{-1}. Cyclic voltammograms are given relative to the Ag/AgCl/KCl (aq saturated) reference electrode.

There is an intense oxidation near zero and little reduction for both complexes (Figures S5 and S6). It is approximately coinciding with silver perchlorate (Figure S7); its voltammogram is needed for confirmation. The presence of this peak in the complexes indicates that there are a lot of free or weakly bounded silver cations.

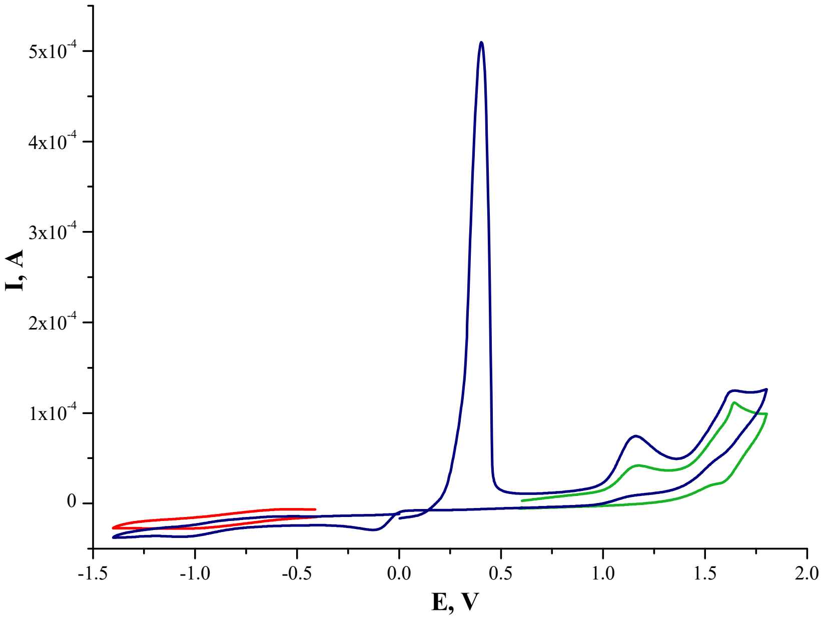

Figure S5. Cyclic voltammogram of AgL1 complex.

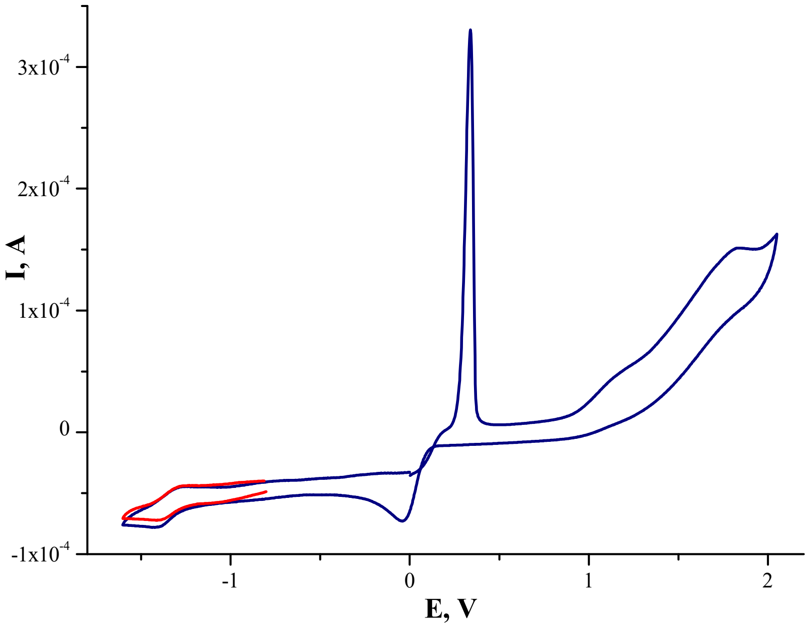

Figure S6. Cyclic voltammogram of AgL2 complex.

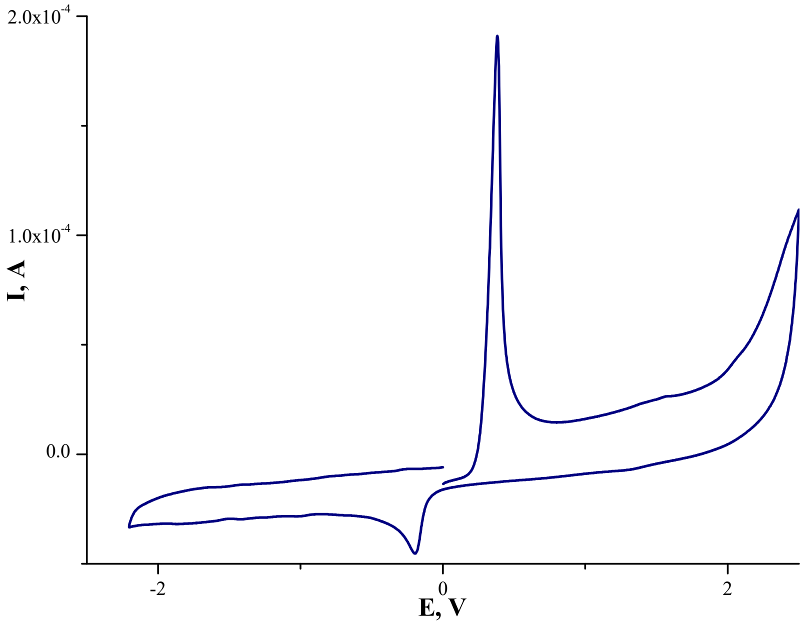

Figure S7. Cyclic voltammogram of $AgClO_4$.
